# Development of Inhalable ATRA-Loaded PLGA Nanoparticles as Host-Directed Immunotherapy against Tuberculosis

**DOI:** 10.3390/pharmaceutics14081745

**Published:** 2022-08-21

**Authors:** Ahmad Z. Bahlool, Sarinj Fattah, Andrew O’Sullivan, Brenton Cavanagh, Ronan MacLoughlin, Joseph Keane, Mary P. O’Sullivan, Sally-Ann Cryan

**Affiliations:** 1 School of Pharmacy and Biomolecular Sciences, Royal College of Surgeons in Ireland (RCSI), 123 St. Stephens Green, D02 YN77 Dublin, Ireland; 2 Tissue Engineering Research Group, Royal College of Surgeons in Ireland (RCSI), 123 St. Stephens Green, D02 YN77 Dublin, Ireland; 3 Department of Clinical Medicine, Trinity Translational Medicine Institute, St. James’s Hospital, Trinity College Dublin, The University of Dublin, D08 9WRT Dublin, Ireland; 4 Research and Development, Science and Emerging Technologies, Aerogen Ltd., Galway Business Park, Dangan, H91 HE94 Galway, Ireland; 5 Cellular and Molecular Imaging Core, Royal College of Surgeons in Ireland RCSI, D02 YN77 Dublin, Ireland; 6 School of Pharmacy and Pharmaceutical Sciences, Trinity College, D02 PN40 Dublin, Ireland; 7 SFI Advanced Materials and Bioengineering Research (AMBER) Centre, RCSI and Trinity College Dublin, D02 PN40 Dublin, Ireland; 8 SFI Centre for Research in Medical Devices (CÚRAM), NUIG & RCSI, H91 W2TY Galway, Ireland

**Keywords:** all trans retinoic acid, ATRA, tuberculosis, host-directed therapy, inhalation, nebulization, pulmonary drug delivery, nanoparticles, PLGA, immunotherapy

## Abstract

Developing new effective treatment strategies to overcome the rise in multi-drug resistant tuberculosis cases (MDR-TB) represents a global challenge. A host-directed therapy (HDT), acting on the host immune response rather than *Mtb* directly, could address these resistance issues. We developed an HDT for targeted TB treatment, using All Trans Retinoic Acid (ATRA)-loaded nanoparticles (NPs) that are suitable for nebulization. Efficacy studies conducted on THP-1 differentiated cells infected with the H37Ra avirulent *Mycobacterium tuberculosis* (*Mtb*) strain, have shown a dose-dependent reduction in H37Ra growth as determined by the BACT/ALERT^®^ system. Confocal microscopy images showed efficient and extensive cellular delivery of ATRA-PLGA NPs into THP-1-derived macrophages. A commercially available vibrating mesh nebulizer was used to generate nanoparticle-loaded droplets with a mass median aerodynamic diameter of 2.13 μm as measured by cascade impaction, and a volumetric median diameter of 4.09 μm as measured by laser diffraction. In an adult breathing simulation experiment, 65.1% of the ATRA PLGA-NP dose was inhaled. This targeted inhaled HDT could offer a new adjunctive TB treatment option that could enhance current dosage regimens leading to better patient prognosis and a decreasing incidence of MDR-TB.

## 1. Introduction

Tuberculosis (TB) is second, only to COVID-19, as the top infectious disease killer globally [1]. According to the 2021 Global TB Report by the World Health Organization (WHO), 5.8 million new cases of TB were diagnosed in 2020, with 214,000 HIV-positive and 1.3 million HIV-negative patients dying from TB in the same year [2]. The current coronavirus pandemic is significantly impacting access to treatment for TB patients and limits case-finding, which has led to increased deaths and a drop in diagnosis, compared with previous years [2].

*Mycobacterium tuberculosis (Mtb)*, the causative microorganism of TB, is phagocytosed mainly by the alveolar macrophages (AM) upon inhalation of contaminated droplets. *Mtb* avoids destruction by blocking phagolysosomal maturation and can persist for long periods of time inside AMs [3,4]. The Th1 host-mediated response leads to containment of infection in the granuloma in healthy individuals, but this does not lead to eradication of the infection [5,6]. In certain cases, such as HIV infection or other causes of immunosuppression, *Mtb* may continue to grow, leading to bacterial spread to broader regions of the lungs and the body, causing active contagious TB [7].

The standard treatment protocol for TB is a multi-drug regimen that lasts for up to 6 months [8]. However, side effects of these drugs and long treatment duration lead to poor patient adherence [9], which has led to multi-drug resistant TB (MDR-TB) [10] and extensive drug resistant (XDR-TB) cases [4]. Thus, new treatment options are needed for TB treatment in order to overcome drug resistance issues and improve dosage regimens, thereby promoting patient adherence.

One approach to boost the TB patient’s immune response is the concept of host-directed therapy (HDT) which targets the immune system of the TB patient—instead of the bacteria—to fight the infection and reduce inflammation-induced tissue damage [11]. Additionally, drug repurposing of currently licensed drugs as HDTs could be considered as a streamlined route for faster drug market access in addition to reducing the drug development-related costs [12,13].

Several case-control studies have showed a relationship between TB and vitamin A deficiency (VAD) [14,15,16,17,18]. Aibana et al. found that household contacts with VAD had a greater than ten-fold increased possibility of contracting TB, with a twenty-fold increase in risk for those aged between 10 and 19 years [19]. However, studies evaluating oral doses of retinol (vitamin A) supplementation have demonstrated that there was no added value for supplementation itself in TB treatment [20,21]. This could be due to increased urinary retinol excretion during infection [22,23], reduced serum carrier proteins level of the proteins which carry retinol to the sites of action [24,25] and down-regulation of the enzymes that are required for the conversion of retinol to all trans retinoic acid (ATRA), which is the active metabolite of vitamin A, in TB patients [26]. Directly targeting ATRA to the lungs as an adjunctive treatment to TB therapy, rather than systemically administering retinol, may sidestep several of these barriers to efficacy.

ATRA is the active metabolite of vitamin A. ATRA, which is also known as Tretinoin, is a licensed medication for treating acute promyelocytic leukaemia (APL) and acne [27]. As previously reported by our lab, human AMs produce ATRA and this suppresses inflammation by induction of FoxP3+ regulatory T-cells which leads to reduced tissue damage caused by excessive inflammation [28]. ATRA exerts its anti-TB effect through several mechanisms; for example, promoting autophagy in *Mtb*-infected human macrophages [29]. Moreover, it is known to deplete certain *Mtb* intracellular nutrients by induction of cholesterol efflux [30,31,32] and to down-regulate transferrin receptors in the cells, leading to a decrease in the supply of iron in phagosomes [33]. In addition, ATRA acts as an immune modulator; it has been shown to modulate the cytokine profile in a rodent model of TB and alter downstream interferon signalling in macrophages in vitro [34,35]. ATRA inhibited the secretion of IL-10 in a 2D in vitro TB model [36], which facilitates phagolysosomal maturation, as previously reported [37]. ATRA also induces functional depletion and maturation of myeloid-derived suppressor cells (MDSCs) which suppress the proliferation of T-cells [38]. ATRA is not directly lethal to *Mtb*; in the absence of macrophages, exposure to ATRA has no effect on bacterial growth compared with a control, indicating that ATRA effects on *Mtb* are based on signalling pathways within the host cell [29].

ATRA is poorly soluble, has a short plasma half-life [39,40], may not reach the targeted tissue in the therapeutic concentration, and can also cause off-target systematic toxicity [41]. Therefore, administration of suitably formulated ATRA locally via the pulmonary route is an attractive approach to achieve a higher drug concentration in the lung. Respiratory drug delivery of ATRA could avoid systemic side effects and target ATRA towards the alveolar macrophages, which are known to take up and clear nanoparticles (NPs) from the lungs following inhalation [42,43,44]. We aim to develop inhalable ATRA-loaded polymeric NPs to deliver ATRA to the lungs and to maximize intracellular uptake of the nanoparticles by macrophages.

## 2. Materials and Methods

### 2.1. Materials

Resomer^®^ RG752H-Poly(D,L-lactide-co-glycolide) (PLGA) acid terminated 4–15 kDa, 75:25 lactide:glycolide ratio, Poly-vinyl alcohol (PVA) 30–70 kDa, 87–90% hydrolyzed, phosphate buffered saline (PBS), All-*trans*-Retinoic Acid (ATRA) ≥98% (HPLC), HPLC grade solvents, Tween^®^ 80, Rhodamine B, Hoechst 33258, Propidium iodide (PI) and Hoechst 33,342 were purchased from Merck-Ireland, (Vale Road, Arklow, Ireland). Decolourizer and Auramine-O stain were bought from Scientific Device Laboratory. HCS CellMask™ Green Stain was purchased from Thermo Fisher Scientific, (Blanchardstown Corporate Park, Ballycoolen, Dublin, Ireland). H37Ra *Mtb* strain and THP-1 cell line were purchased from the American Type Culture Collection (ATCC, Manassas, VA, U.S.A.). Materials needed to prepare Middlebrook 7H9 medium were obtained from BD. RPMI 1640 and Gibco™ Fetal Bovine Serum were purchased from Bio-Sciences.

### 2.2. Methods

#### 2.2.1. Formulation of ATRA-PLGA NPs Using Nanoprecipitation

ATRA (1 mg) and PLGA (20 mg) were dissolved in 1 mL of acetone which was then added dropwise, under magnetic stirring, to 3 mL of PVA aqueous solution (2% *w*/*v*) at room temperature and left overnight to achieve a stable colloidal suspension and allow the acetone to evaporate. The emulsion was centrifuged, at 13,000 rpm for 25 min, and washed two times with deionized water in order to remove excess PVA and the non-encapsulated drug. For Rhodamine B labelled formulations, 100 µg of Rhodamine B were added to the organic phase. Samples were collected before and after centrifugation to determine ATRA encapsulation efficiency. Several manufacturing parameters were optimized to produce particles of the desired size including polymer to drug *w*/*w* ratio and concentration of the polymer and drug in the organic phase (Supplementary Table S1).

#### 2.2.2. Physicochemical Characterization of Nanoparticles

Measurements of the hydrodynamic diameter (Z-average), polydispersity index (PDI) and zeta potential (ZP) were performed by dynamic light scattering (DLS), and electrophoretic mobility by Malvern Nanosizer ZS90. The nanoparticles were diluted in 0.1x PBS at 1:100 (*v*/*v*) ratio for both size and zeta potential at pH 7.4. Electrophoretic mobility was used to determine the ZP using the Smoluchowski equation. All measurements were performed at 25 °C in triplicate and mean ± standard deviation (SD) were recorded.

#### 2.2.3. Morphology of ATRA-PLGA NPs

To visualize the nanoparticles using transmission electron microscopy (TEM), NPs were diluted 1:100% *v*/*v* in distilled water corresponding to 0.1 mg/mL of the PLGA. An amount of 5 µL of the ATRA-PLGA was added on a 200 mesh formvar and silicone monoxide coated copper grid (Ted Pella Inc., Mountain Lakes Blvd Redding, CA, USA) and dried overnight. The grid was stained with 5 µL of Uranyl Acetate Alternative (Ted Pella Inc., Redding, CA, USA) for one minute. Excess stain was removed to yield a dry grid. Image acquisition was performed using a Hitachi H7650 transmission electron microscope (TEM) (Tokyo, Japan) operating at 100 kV with a side mounted camera.

#### 2.2.4. Encapsulation Efficiency and Drug Loading

ATRA-PLGA NPs (10 µL) were dissolved 1:100 (*v*/*v*) in methanol. Then, particles were vortexed to ensure complete disruption. ATRA concentration was quantified by measuring the absorbance by UV-Vis spectrophotometer at 348 nm. Encapsulation efficiency (EE) and drug loading (DL) of ATRA were calculated as below:

$$\begin{array}{l} \mathrm{Encapsulation}\;\mathrm{efficiency}\;\%\;\\ =(Conc.\;of\;ATRA\;after\;purification/Conc.\;of\;ATRA\;before\;purifaction)\times 100\%\; \end{array}$$

Drug loading % =(Mass of encapsulated drug/Mass of the polymer)×100%

#### 2.2.5. Cell Culture of THP-1 Cell Lines

Cells were cultured in T-75 vented flasks with complete RPMI 1640 (cRPMI) supplemented with 10% fetal bovine serum (FBS). For differentiation, the THP-1 cells were centrifuged at 1200 rpm for 5 min in a 50 mL tube at 21 °C. After discarding the supernatant, the pellet was re-suspended with the media and counted. Then, THP-1 cells were seeded at a density of 1 × 10^5^ cells/mL in Nunc plates (12, 48 or 96-wells depending on aim of analysis). Cells were differentiated into adherent macrophages for 72 h at 37 °C and 5% CO_2_ using 100 nM Phorbol 12-myristate 13-acetate (PMA). Individual plates were set up for each time point during the experiment. To determine multiplicity of infection (MOI), THP-1 cells were also seeded in glass 8-well chamber Nunc Lab-Tek II slides at the same density (Fisher scientific, Dublin, Ireland).

#### 2.2.6. Nanoparticle In Vitro Cell Uptake Study

To visualize cell uptake of NPs, cells were seeded on coverslips and differentiated with PMA for 72 h in a 24-well plate. Then, cells were incubated for 24 h with cell media, ATRA-Rhodamine B co-loaded NPs (corresponding to 5 μg/mL of ATRA) and blank NPs (corresponding to ATRA-Rhodamine co-loaded NPs). Then, the media was removed, and the cells were washed with PBS twice and then fixed for 15 min using 4% paraformaldehyde (PFA). After removal of the PFA solution, HCS CellMask™ Green (2 μg/mL) was added to stain cytoplasm and Hoechst 33,342 (4 μg/mL) was added to stain nuclei. The coverslips were mounted on a microscope glass slide. Images were taken using a Carl Zeiss LSM 710 confocal microscope (Carl Zeiss, Jena, Germany), equipped with a Plan-Apochromat 40× (NA 1.4) with 0.37 µm inter-slice Z spacing to yield a total image Z depth covering the complete cell. Images were recorded at 1320 × 1320 pixels and 0.39 µs dwell time. Z stack images were prepared in FIJI and were maximum intensity projected [45].

#### 2.2.7. *Mtb* Culture and Cell Infection

For macrophages infection, the H37Ra *Mtb* avirulent strain was used. Experiments with H37Ra were performed in a level II biosafety cabinet (BSC class 2). ATRA was used in dark conditions during the experiments. H37Ra *Mtb* was propagated to log phase in Middlebrook 7H9 at 37 °C. The H37Ra was centrifuged for 10 min at 3800 rpm. The bacterial pellet was re-suspended using RPMI and de-clumped with a 25G needle. Then, to pellet any remaining clumps, the bacterial suspension was centrifuged at 800 rpm for 3 min. The upper 75% by volume was used to avoid bacterial clumps. Quantities of 5, 10, 15, 20, 25 and 30 μL of the bacteria were added to the chambers of the Lab-Tek II slides. The slides were incubated for 3 h at 5% CO_2_ and 37 °C.

Determination of number of bacteria per cell (MOI) is important to have controlled conditions across all experiments. To determine MOI, the infected THP-1 cells were fixed using 4% Paraformaldehyde (PFA) within the Lab-Tek II slides for 10 min. Then, the PFA was removed and the *Mtb* were stained with Auramine-O followed by the Decolorizer. The nuclei were stained with Hoescht 33,342 and DAKO anti-fade was added. The slides were examined under a 100× oil objective lens using Olympus IX51 fluorescent microscope to assess the extent of infection in each chamber. Under the FITC filter, Mycobacteria fluoresce green, and cell nuclei fluoresce blue under the DAPI filter. The required volume of level of infection of *Mtb* suspension for the desired MOI was calculated by determining the ratio of surface area between the Nunc 12-well plates’ well and Lab-Tek II chamber well. The calculated volume was added to the 12-well plate wells and then the plates were incubated for 3 h at 5% CO_2_ and 37 °C to allow the phagocytosis of the mycobacteria. The wells were washed after 3 h with fresh media to remove any extracellular mycobacteria and then treatment conditions were added.

#### 2.2.8. Assessment of Bacterial Growth following Treatment Using the BacT/ALERT^®^ System

The protocol of using the BacT/Alert^®^ system for preclinical testing of HDT for TB has previously been described by our group [46]. Briefly, ATRA-PLGA NPs and ATRA solution were added to the wells of THP-1 cells infected with H37Ra at the following concentrations: (5, 10, 15, 20 μg/mL of ATRA) for 72 and 120 h. Then the medium was removed from each well and transferred to conical tubes. Lysis buffer consisting of 500 μL of Triton-X 100 (0.1% *v*/*v*) in PBS was added for 15 min at room temperature to each well to lyse the cells. Then, cells were collected from the plate with a cell scraper and added to their supernatant. Each well was washed with 500 μL of sterile PBS and was added to its corresponding supernatant. The suspension of each sample was mixed and de-clumped by using a 25G needle. An amount of 1:10 dilution of each suspension—in Middlebrook—were prepared and 500 μL of the bacterial suspension were added along with 500 μL mycobacterial antibiotic supplement to the BacT/ALERT^®^ MP bottles (bioMérieux, Basingstoke, UK). The bottles were placed in the BacT/ALERT^®^ device (bioMérieux, UK). This automated liquid culture system allowed determination of the viability of microorganisms via a colorimetric sensor that is sensitive to CO_2_ levels in the culture media, thus, detecting the time to positivity (TTP) over a 42-day period following treatment. The TTP was used to calculate the change in bacterial growth (%) in response to treatment based on the level of infection present at 3 h, as shown below:

Change in bacterial growth %=((TTP Day 0−TTP Day 3)/TTP Day 0)×100%

#### 2.2.9. Determination of Cell Viability Using the MTS Assay

The cytotoxicity of ATRA solution (Free ATRA) and ATRA- PLGA NPs, in vitro, were assessed in THP-1 cells by measuring metabolic activity using MTS assay. Briefly, in a 96-well plate, 200 µL of 1 × 10^5^ cells/mL were seeded in 100 nM PMA for 72 h followed by 24 h in absence of PMA at 37 °C and 5% CO_2_. On day 4, the cells were treated with ATRA-PLGA NPs or ATRA solution at (5, 10, 15, 20 µg/mL of ATRA) and further incubated for 24 h and 72 h. An amount of 10% DMSO was used as positive control. CellTiter 96^®^ Aqueous One Solution (Promega, Madison, WI, USA) (20 µL) was then added to each well and incubated for 2 h. Results were recorded using the Tecan Pro 100 Plate Reader, under the setting of absorbance at 490 nm. The relative cell metabolic activity was expressed as a percentile of treated to untreated cells.

#### 2.2.10. Estimating Cell Viability Using Propidium Iodide (PI) Based Cell Exclusion Assay

Differentiated THP-1 in 48-well plates were treated with Free ATRA or ATRA-PLGA NPs (5, 10, 15, 20 µg/mL of ATRA). THP-1 cell viability was assessed using a PI cell exclusion assay. THP-1 cells were stained for 10 min at room temperature with 50 µg/mL Hoechst 33258, 5 µg/mL PI, and 20 µg/mL Hoechst 33,342 in dark conditions. Dead cells were determined via PI staining positivity (red) and total number of cells were identified via blue nuclei Hoechst staining using a high content analysis (HCA) imaging system (Lionheart™ FX (Bio-Tek, Winooski, VT, USA). Four view fields were acquired per well.

%PI positive(dead)=(Number of PI positivedead cells (red)/Total number of cells (blue))×100%

#### 2.2.11. Characterization of Aerosol Droplet Size

ATRA-PLGA NPs were aerosolized with a vibrating mesh nebulizer (VMN, Dulacca St, Acacia Ridge, Australia) (Aerogen Solo^®^) in combination with a valved holding chamber and mouthpiece (Aerogen Ultra, Aerogen Ltd., Galway, Ireland). ATRA-PLGA NPs were suspended in saline (0.9% NaCl) as per the typical formulations for inhalation. Per US FDA guidance for nebulizers, the generated droplet size of the aerosol was determined using laser diffraction technique (Spraytec, Malvern, UK) and cascade impaction at 15 liters per minute (LPM) (W7, Westech, UK). All testing was performed in triplicate (n = 3).

##### Laser Diffraction

Droplet size as volume median diameter (VMD) and the respirable fraction of the ATRA-PLGA NPs (10 mg/mL) were determined as previously described [47] using Spraytec with RT sizer software. The Aerogen Solo/Ultra plus mouthpiece was connected to the droplet sizer inlet and run until all the dose (0.25 mL) was delivered.

##### Cascade Impaction

The mass median aerodynamic diameter (MMAD) and the geometric standard deviation (GSD) were determined by W7 cascade impactor (W7, Westech) at 15 LPM. The Aerogen Solo/Ultra plus mouthpiece was connected to the cascade impaction induction port and turned on until the entire dose (1 mL) was delivered. The time taken to deliver the dose was 3.5 min. The Aerogen Solo VMN was weighed before and after nebulization to determine the residual drug substance remaining unnebulized in the medication cup. The mass of drug substance collected on each of the components was eluted with 5 mL of methanol and quantified using UV-Vis spectrophotometer at 348 nm. The MMAD was determined by calculating the median cumulative drug mass percentages of deposited ATRA on the W7 apparatus stages, which was plotted against the corresponding stage diameter cutoff value and then a log-linear on 50% cumulative mass was applied to determine the median. Square root of the particle size distribution at 84th percentile and 16th percentile were used to calculate GSD.

Fine particle fraction (FPF)% was identified as the collected ATRA from stage 3 to the micro-orifice (MOC) filter of the cascade impactor. FPF can be expressed as emitted dose (FPF_ED_) or nominal dose (FPF_ND_), where the former describes the FPF ratio to the mass that reached stage 1 and below, and the latter represents the FPF ratio to the dose loaded in the nebulizer before impaction:

$$FPF_{ED}=\left(Sum\;of\;stages\;3-7/amount\;recovered\;on\;stages\;1-8\right)\times 100\%$$

$$FPF_{ND}=\left(Sum\;of\;stages\;3-7/amount\;loaded\;on\;the\;device\right)\times 100\%$$

#### 2.2.12. Effect of Nebulization on the Physicochemical Properties of Nanoparticles

To assess the effect of the nebulization on the physicochemical characteristics of the ATRA-PLGA NPs, the nanoparticles were suspended in saline and nebulized using the Aerogen Solo vibrating mesh nebulizer into a 50 mL falcon tube. The physicochemical properties (particle size and PDI) were measured using a Zetasizer, as previously described in Section 2.2.2.

#### 2.2.13. Assessment of Inhaled Dose to a Simulated Patient

**Figure 1:**
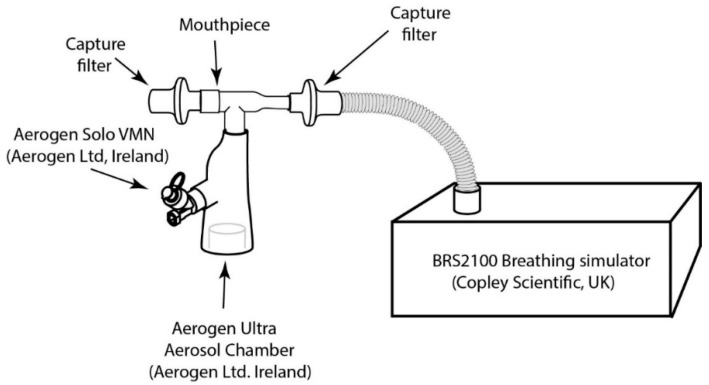
Inhaled dose assessment of nebulized ATRA-loaded PLGA NPs experiment set up.

In accordance with USP 1601 [48] and Ph.Eur.2.9.44 [49], using a BRS 2100 breathing stimulator (Copley Scientific Ltd., Colwick, UK) an adult breathing profile was generated (inhalation to exhalation (I:E) ratio: 1:1, tidal volume: 500 mL, waveform: sinusoidal and frequency: 15 breaths per minute (BPM)) to assess the inhaled dose. The breath was confirmed using a gas flow analyzer, CITRIX H5 (IMT Analytics, Buchs, Switzerland). The Aerogen Solo/Ultra plus mouthpiece was connected via a capture filter (RespirGard II 303EU, Vyaire, Mettawa, IL, USA) to a breathing stimulator. No supplemental oxygen was applied through the Ultra chamber. Dosing was performed using 1 mL of ATRA-PLGA NPs suspended in saline. The mass of drug substance collected on each capture filter was eluted with 9 mL of methanol and quantified using a UV-Vis spectrophotometer. All testing was completed in triplicate (n = 3). The set-up of the experiment is shown in Figure 1, below.

#### 2.2.14. Statistical Analysis

GraphPad Prism 9 software was used for statistical analysis. Two-way ANOVA tests and Tukey’s post hoc analysis were used to identify differences between experimental groups. * *p* < 0.05, ** *p* < 0.01, *** *p* < 0.001, and **** *p* < 0.0001.

## 3. Results

### 3.1. Formulation and Characterization of ATRA-PLGA NPs

**Figure 2:**
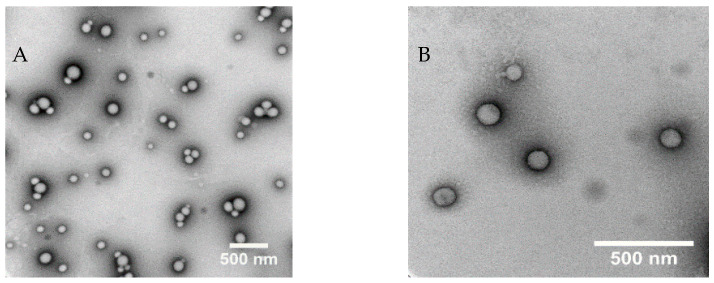
TEM image of ATRA-PLGA NPs corresponding to 0.1 mg/mL of PLGA. Image acquisition using a Hitachi H7650 TEM (Hitachi, Chiyoda, Tokyo, Japan) operating at 100 kV with a side mounted camera at a magnification of (**A**) 40,000× and (**B**) 100,000×. Scale bar: 500 nm.

The hydrophobic PLGA polymer forms nanoparticles based on the solubility difference of PLGA in acetone, in comparison with the PVA aqueous phase (non-solvent) which creates a supersaturated mixture leading to PLGA precipitation [50]. In this study, we used acetone as an organic solvent, due to its low toxic potential to human health (Class III in ICH Q3C) [51]. Our results show that increasing the drug to polymer ratio (*w*/*w*) led to increases in size and polydispersity index (PDI). In addition, drug loading was increased as more drug was added (Supplementary Table S1). An amount of 1:20 drug: polymer (*w*/*w*) ratio was chosen for further testing due to the preferable physicochemical properties and encapsulation efficiency achieved with this formulation (Table 1). TEM imaging showed that the particles have a spherical shape (Figure 2).

### 3.2. Cell Uptake Study

**Figure 3:**
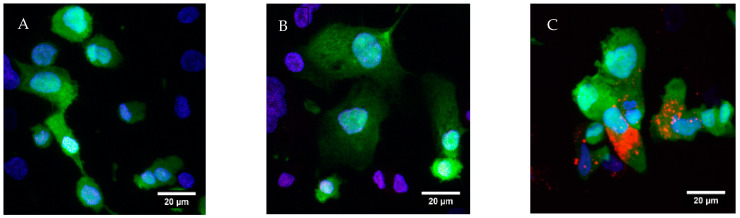
Confocal microscopy images of cellular uptake and distribution of ATRA-Rhodamine co-loaded PLGA NPs treatment for 24h. THP-1 differentiated macrophages were treated with (**A**) RPMI, (**B**) blank PLGA NPs, and (**C**) ATRA-Rhodamine co-loaded PLGA NPs (red) and viewed 24 h later using confocal microscopy (Carl Zeiss, Jena, Germany) at 40×. Hoechst (blue) stains nuclei, HCS CellMask™ Green (green) stains cytoplasm. Scale bar: 20 µm.

Since alveolar macrophages are the niche for *Mycobacterium tuberculosis* [52], we used THP-1 derived macrophages as a model to assess the ability of our nanoparticles to be taken up by the cells in order to deliver ATRA to the site of action. Cells were treated with either RPMI (Figure 3A), blank PLGA NPs (Figure 3B), or ATRA-Rhodamine B co-loaded PLGA NPs (Figure 3C). After 24 h of treatment, extensive cellular uptake of the ATRA-Rhodamine B PLGA nanoparticles (red dots) by differentiated THP-1 cells was observed. The nanoparticles showed cellular distribution within the cell cytoplasm, which was evident as co-localization, with the green colour as shown (Figure 3C).

### 3.3. Effect of ATRA Treatment on H37Ra Growth and Cell Morphology In Vitro

**Figure 4:**
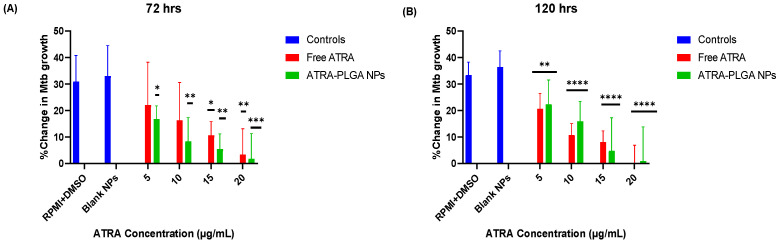
In vitro treatment with ATRA reduced the growth of H37Ra *Mtb*. Baseline infection levels of THP-1 cells infected with H37Ra were measured at 3 h post-infection followed by cells treatment. Treatment groups were RPMI and DMSO (DMSO at 0.4% *v/v*-corresponding to the highest volume used with free ATRA), Free ATRA solution (5, 10, 15, 20 µg/mL), unloaded-PLGA NPs (blank NPs), and ATRA-PLGA NPs (equivalent to 5, 10, 15, 20 µg/mL of ATRA). Treatment efficacy was assessed at (**A**) 72 h, and (**B**) 120 h, after treatment using the BacT/Alert^®^ 3D culture system (bioMerieux) by calculating the % change in bacterial growth. MOI: 1–10 per cell (n = 3). Statistical analysis was performed using two-way ANOVA with Tukey’s post-hoc test comparing RPMI and DMSO, and blank NPs groups as reference. * *p* < 0.05, ** *p* < 0.01, *** *p* < 0.001, and **** *p* < 0.0001.

Free ATRA and ATRA-PLGA NPs reduced bacterial growth significantly at 72 h and 120 h post-treatment, in comparison with untreated controls (Figure 4). No significant difference between free ATRA (ATRA solution) and ATRA-PLGA NPs was observed at both time points (Figure 4). The 120 h treated cells (Figure 4B) showed more statistical significance in reducing bacterial growth compared with 72 h treated cells (Figure 4A).

**Figure 5:**
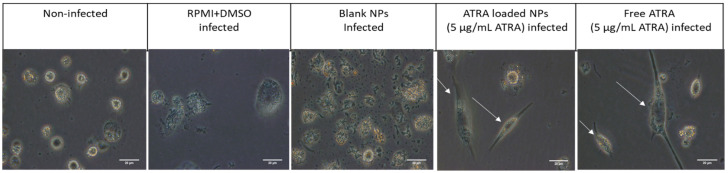
Fluorescent microscope images of cell morphology after 72 h of ATRA treatment (5 µg/mL). THP-1 cells infected with H37Ra *Mtb* strain were treated with RPMI and DMSO, unloaded-NPs (blank NPs), 5 μg/mL of free ATRA solution and ATRA-PLGA NPs (corresponding to 5 μg/mL ATRA). Scale bar: 20 µm. Arrows point at elongated macrophages.

Cells treated with both ATRA solution and ATRA-PLGA NPs showed a different morphology as they became elongated in comparison with blank NPs, RPMI and DMSO treated and non-infected non-treated cells which showed a circular shape (Figure 5).

### 3.4. Effect of ATRA Treatments on Cell Viability

**Figure 6:**
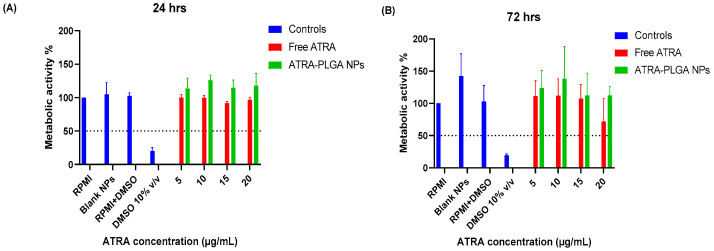
ATRA treatment effect on cell viability as determined by MTS assay. MTS assay was performed at (**A**) 24 and (**B**) 72 h in the absence of H37Ra, and post-treatment of THP-1 cells with ATRA solution in DMSO and ATRA-PLGA NPs (5, 10, 15, 20 µg/mL of ATRA). RPMI and DMSO 10% *v/v* groups were utilized as the negative and positive controls, respectively. Results were plotted as (%) metabolic activity relative to RPMI group. Data shown as mean ± SD (n = 3). Statistical analysis was conducted using two-way ANOVA with Tukey’s post-hoc test.

The cell viability of differentiated THP-1 cells after ATRA treatment was determined by an MTS assay. Treatment of THP-1 cells with free ATRA solution or ATRA-PLGA NPs did not affect cell viability in comparison with controls (RPMI alone, RPMI and DMSO (DMSO at 0.4% *v/v*—corresponding to the highest volume used with free ATRA), and blank NPs) after 24 h and 72 h of treatment and all used doses (5, 10, 15, 20 µg/mL) were above the 50th percentile cell viability limit of toxicity (Figure 6A,B). However, there was a small decrease in cell viability on the highest dose of free ATRA at 72 h but it was not statistically significant (Figure 6B).

**Figure 7:**
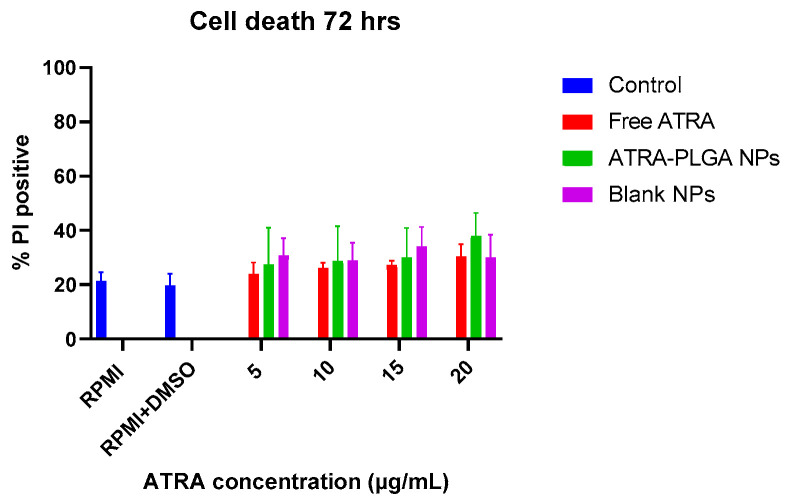
The effect of ATRA treatment on cell death. THP-1 cells were treated with free ATRA, ATRA-PLGA NPs (5, 10, 15, 20 µg/mL of ATRA) and blank PLGA NPs (equivalent to ATRA-PLGA NPs doses). PI based cell exclusion assay was used to measure cell viability. Cells were stained with 20 µg/mL Hoechst 33,342, 5 µg/mL PI, and 50 µg/mL Hoechst 33,258 for 10 min under dark conditions. Total cell counts were identified via Hoechst staining of nuclei (blue) and dead cells were determined via PI staining (red), by using the Lionheart™ FX Automated Microscope. Data were plotted as (%) of PI positive dead cells over the total number of cells. Four view fields were acquired per well. Data shown as mean ± SD (n = 3). Statistical analysis was conducted using two-way ANOVA with Tukey’s post-hoc test.

The in vitro cytotoxicity study of ATRA on non-infected THP-1 cells was also determined by Propidium Iodide (PI) exclusion assay after 72 h to confirm the results of the MTS assay. The percentage of PI positive cells after ATRA solution and ATRA-PLGA NPs treatment at (5, 10, 15, 20 µg/mL of ATRA) did not indicate statistically significant cell death in comparison with controls (RPMI, RPMI and DMSO (DMSO at 0.4% *v/v*—corresponding to the highest volume used with free ATRA), and blank NPs (equivalent doses to ATRA-PLGA NPs)) (Figure 7).

### 3.5. Aerosol Droplet Size Analysis

**Figure 8:**
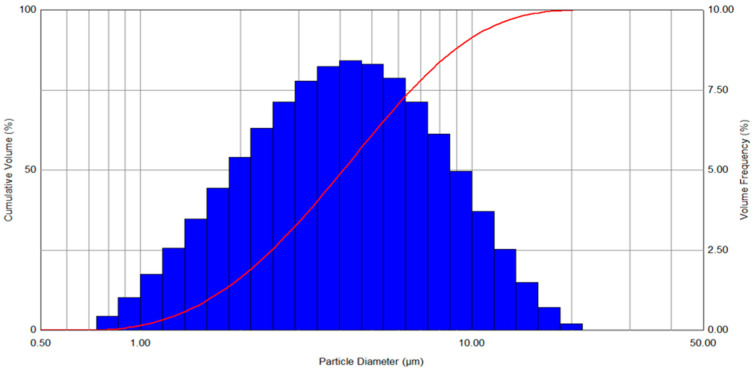
Particle size distribution of nebulized droplets of ATRA-PLGA nanoparticles aerosolized using Aerogen Solo VMN as measured by laser diffraction via Malvern Spraytec.

ATRA-PLGA NPs were nebulized with Aerogen Solo VMN in order to deliver it via inhalation. The generated aerosol droplets contain the suspended nanoparticles in order to deliver them to the lung. We have studied the aerodynamic properties of the generated aerosol and the potential distribution in the lung. The volumetric mean diameter (VMD) of the ATRA-PLGA NPs was 4.09 ± 0.1 μm and the FPF(%) was 60.88 ± 1.51%, as characterized by laser diffraction (Figure 8 and Table 2).

**Figure 9:**
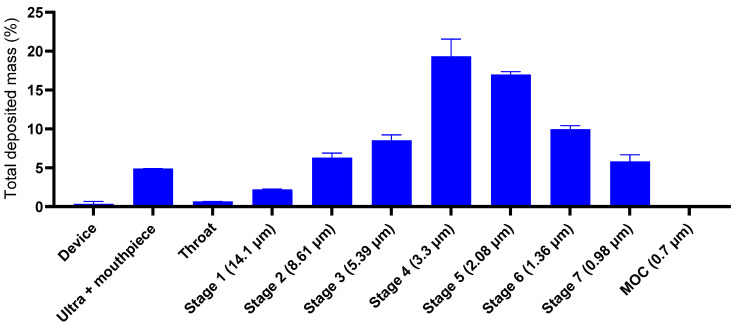
Quantification of the deposited ATRA-PLGA NPs. Mass distribution of nebulized nanoparticles using cascade impactor at 15 LPM (W7, Westech, UK). The mass of drug substance collected on each of the components was eluted with 5 mL of methanol and quantified using a UV-Vis spectrophotometer at 348 nm. Results are shown as the % total drug deposited on all stages of the impactor including the throat. The data are represented as nominal dose mass percentage. Data are shown as mean ± SD (n = 3).

The nebulized ATRA-PLGA NPs MMAD as evaluated by cascade impaction was 2.13 ± 0.057 μm with GSD of 1.93 ± 0.041, FPF_ND_ (%) 64.19 ± 6.9, and FPF_ED_ (%) 81.81 ± 1.16, as summarized in Table 3 below. Separation of the nebulized ATRA-PLGA NPs into fractions with different sizes was based on the aerodynamic mass. Quantification of each size fraction of deposited ATRA-PLGA NPs showed that the majority of ATRA-PLGA NPs were deposited on the stages of 3, 4, 5 and 6, indicating a range of droplet size between approximately 1 to 5 μm for the majority of nebulized ATRA-PLGA NPs (Figure 9).

To determine the nebulization effect on the physicochemical characteristics of the ATRA-PLGA NPs, nanoparticles were nebulized into a 50 mL falcon tube and then samples were collected. The average hydrodynamic size and PDI of ATRA-PLGA NPs were determined by the Malvern Nanosizer ZS-90 (Malvern, UK). The nebulization process of ATRA-PLGA NPs suspended in saline led to a slight increase in particle size from 264.7 ± 8.6 nm to 316.7 ± 12.01 nm, and PDI from 0.22 ± 0.008 to 0.31 ± 0.027. No change in zeta potential was observed.

### 3.6. Inhaled Dose Assessment in Patient Simulated Breathing Profile

The percentage of nebulised ATRA-PLGA NPs delivered to a simulated spontaneously breathing patient using the Aerogen Solo/Ultra plus mouthpiece was assessed. In a simulated adult spontaneous breathing patient, 65.11 ± 0.61% (of nominal dose) was delivered at the exit of the mouthpiece, i.e., the expected inhaled dose, with a supplemental oxygen flow rate of 0 LPM.

## 4. Discussion

New drug delivery approaches that harness medical devices and advanced formulation to allow for precise drug targeting to the lungs and alveolar macrophages have gained much attention in recent years [53,54,55]. However, under the current regulations, new chemical molecules may take up to 15 years to obtain market authorization. Therefore, repurposing of currently available drugs in the market could be a streamlined route to shorten the long timeline that patients with TB globally cannot afford. An inhalable macrophage-targeting drug delivery system that could increase efficacy, reduce the side effects associated with conventional therapies, and improve adherence rates and dosage regimen is of huge clinical interest.

PLGA is an FDA approved polymer that is generally synthesized by ring opening polymerization [56]. PLGA is widely used in drug delivery due to its biocompatibility, biodegradability, and the ability to formulate it in several forms to meet the goal of the formulation [57]. It can also be used to encapsulate a wide range of substances such as small molecules, proteins, nucleic acids and vaccines [58]. Several studies have focused on PLGA as a polymeric antibiotics drug delivery system [36,59,60]. Previously published data indicate that the carrier itself (PLGA) could affect immune cell function [59]. A synergistic effect of PLGA microparticles and LPS signaling in dendritic cells (DCs) has been observed in TLR-mediated inflammasome activity [61]. In addition, ATRA-PLGA microparticles decreased transcription of iNOS and TNF-α in mouse TB model lung homogenates in comparison with ATRA alone [36]. In an acute lung injury model, iNOS decrease is associated with improved disease pathology [62]. However, these microparticles face issues of scalability and difficulty in terms of integration with many common aerosol delivery systems including nebulizers. Therefore, we thought to combine PLGA nanoparticle drug delivery system with ATRA as an HDT to beneficially modulate the immune system and enable integration with vibrating mesh nebulizer devices [63,64].

We successfully formulated ATRA into PLGA NPs with a size of 261.6 ± 9.7 nm and encapsulation efficiency of 65.8 ± 12.4% using the nanoprecipitation method. Our in vitro testing indicated a good uptake of the nanoparticles and a cytoplasmic distribution of the particles within macrophage-like differentiated THP-1 cells (Figure 3). In addition, free ATRA solution and ATRA-NPs led to significant reduction of the growth of H37Ra *Mtb* in THP-1 infected cells in a dose dependent manner (Figure 4). One of the early reports (published in 1989) of retinoic acids’ role in TB showed that it was able to slow or stop intracellular *Mtb* bacterial replication in human macrophages at a dose of 1 × 10^−5^ M [65]. Wheelwright et al. also demonstrated reduced bacterial burden in monocytes and monocyte-derived macrophages (MDMs) infected with H37Ra and H37Rv strains treated with ATRA at 1 × 10^−8^ M along with reducing cholesterol which is an important bacterial intracellular nutrient [32,66].

A good balance between the anti-inflammatory and pro-inflammatory immune responses is required to control *Mtb* infection within the host. A previous report showed that ATRA treatment (2 µM) for 3 days promoted M1 to M2 differentiation in murine bone marrow derived macrophages [67]. In our hands, ATRA treatment induced cell elongation (Figure 5) which is associated with M2 polarization of in vitro treated macrophages and could indicate a pro-healing anti-inflammatory phenotype which could help in reducing tissue damage in the lung during TB infection [68,69].

Cell viability studies conducted using MTS assay and PI exclusion assay indicate that ATRA treatment (free ATRA or ATRA-PLGA NPs) at doses (5, 10, 15, 20 µg/mL) that were able to decrease *Mtb* growth, did not lead to significant host cell death (Figure 6 and Figure 7). However, further investigation on other cell types such as lung epithelium and fibroblasts will be required to further confirm the biocompatibility of the formulation.

Pulmonary drug delivery leads to less systemic toxicity and offers an opportunity to deliver a high drug dose to the lungs [36,70]. Inhaled Rifampicin delivered to pigs’ lungs showed increased concentration in comparison with other administration routes [71]. ATRA cannot easily be aerosolized because of its lipophilicity. Thus, its formulation and encapsulation into a nanoparticle drug delivery system offers a means to effectively aerosolize ATRA to target the lungs and thereby reach a higher dose locally in the lung compared with systemic administration, and in addition, facilitate its uptake by the *Mtb*-infected cells.

Vibrating mesh nebulizers have showed enhanced drug delivery efficiency compared with jet nebulizers, with shorter treatment duration, minimal residual volume, and generation of fine particle fractions which allows delivery to the peripheries of the lung [72,73,74]. Compared with jet nebulizers, the Aerogen Solo is able to deliver more medication to the lungs [75] in less time [76]. Droplet size of aerosols has an important role in increasing delivery to the targeted site of the respiratory tract [77]. Aerodynamic size range of droplets and particles from 1–5 μm are shown to be optimal for pulmonary drug delivery [78,79,80]. The VMD of the ATRA-PLGA NP formulation aerosolized using Aerogen Solo VMN was measured to be 4.09 ± 0.01 µm (Table 2, Figure 8) which is considered appropriate for lung deposition [81]. The MMAD of the nebulized ATRA-NP formulation was 2.13 ± 0.057 µm (Table 3, Figure 9). This MMAD would be predictive of significant drug deposition in the distal areas of the respiratory tract. Desai et al. prepared ATRA-loaded niosomes for use in targeted lung cancer treatment. They nebulized the niosomes (1 mg of ATRA/mL) with a jet nebulizer and evaluated the properties of the generated aerosol; their results showed an encapsulation efficiency above 50% for their formulations, and MMAD of 3.7 and 3.58 µm as measured by an Anderson cascade impactor [82].

Although nebulization of ATRA-PLGA NPs has led to a slight increase in the NPs’ size and PDI, they are still in the range to allow effective uptake by macrophages [83]. We hypothesize that this increase in size is due to the presence of salt (0.9% *w/v* NaCl) in the nanoparticles suspension as per typical formulations for inhalation. To confirm this, we undertook the same experiment with water instead of saline as a suspending media, and no increase in size or PDI of the ATRA-PLGA NPs was observed (Supplementary Figure S1).

In order to predict in vivo aerosol performance, the inhaled dose delivered during a simulated normal adult breathing pattern was conducted, and was found to be 65.11 ± 0.61%. Elena et al. reported a simulation experiment in which they used an adult 3D printed head model that was connected to a breathing simulator at the trachea bifurcation level using a capture filter. At the level of the trachea, the mouthpiece-mediated VMN aerosol delivery delivered 29.02 ± 1.41% of the nominal dose [84]. More in-depth simulation experiments using different head and lung models will be needed to further investigate the effect of breathing patterns on the delivered dose of ATRA-PLGA NPs, in order to obtain a fuller appreciation of the potential spread in inhaled doses.

## 5. Conclusions and Future Perspectives

This work is based on existing literature about the benefits of using ATRA as a host-directed therapy treatment by developing a new ATRA formulation, in the form of NPs suitable for inhalation, from manufacturing through to in vitro pre-clinical testing. This HDT treatment for TB is a promising targeted approach that could help to improve current treatments aiming for better prognosis for patients, and to reduce MDR-TB incidence. More information is emerging on ATRA as a potential HDT [85].

Future work will aim to scale up this formulation using microfluidics and perform in vivo efficacy studies. The immune response of the host to TB infection is complicated, and some host functions are considered vital at early stages, but, at late stages, can be detrimental [86]. In the personalized medicine era, genetic variation should also be considered as it may impact the response to HDT [87,88]. Thus, understanding the HDT’s mechanisms of action and how they interact with the immune system of the host is very important in considering them properly and avoiding an underestimation of their efficacy. Detailed endotypic and phenotyping characterization of these trial patients could help identify which patients may benefit from HDT treatment [89,90].

## Figures and Tables

**Table 1:** Physicochemical properties, encapsulation efficiency and drug loading of ATRA-PLGA NPs; nanoparticles were formulated using the nanoprecipitation method (1:20 *w*/*w* drug:polymer). All loaded formulations were purified using centrifugation, disrupted in 1:100 (*v*/*v*) NPs/MeOH. UV-Vis was used to quantify the drug content at 348 nm. The average hydrodynamic diameter, PDI and zeta potential of NPs were measured by a Malvern Nanosizer ZS-90. Data reported as mean ± SD (n = 3). EE: encapsulation efficiency, PDI: polydispersity index.

Formula	Size (nm)	PDI	Zeta Potential (mV)	EE%	Drug Loading (µg/mg)
Blank NPs	203.9 ± 0.4	0.041 ± 0.006	−2.52 ± 0.331	N/A	N/A
ATRA-PLGA NPs	261.6 ± 9.7	0.187 ± 0.030	−1.80 ± 0.410	65.8 ± 12.4	33.8 ± 4.56

**Table 2:** Aerodynamic properties of the aerosolized ATRA-PLGA NPs aerosolized using Aerogen Solo VMN as measured by laser diffraction via Malvern Spraytec. Results are representative of three independent nebulization runs (n = 3), mean ± SD. VMD = volumetric median diameter, FPF = fine particle fraction.

Dv10 (μm)	Dv50 (VMD) (μm)	Dv90 (μm)	FPF (%) < 5µm	Flow Rate (mL/min)
1.62 ± 0.02	4.09 ± 0.01	9.7 ± 0.46	60.88 ± 1.51	0.28 ± 0.03

**Table 3:** ATRA-PLGA nanoparticles aerodynamic properties. Particles were nebulized with Aerogen Solo VMN. Results are shown for three independent nebulization runs (n = 3), mean ± SD, of nanoparticles suspended in 1 mL of saline solution. MMAD: mass median aerodynamic diameter; GSD: geometric standard deviation; FPF_ND_: fine particle fraction of nominal dose; FPF_ED_: fine particle fraction of emitted dose.

MMAD (μm)	GSD	FPF_ND_ (%)	FPF_ED_ (%)	Mass Balance (%)
2.13 ± 0.057	1.93 ± 0.041	58.59 ± 6.29	81.81 ± 1.162	88.5

## Data Availability

The data is available upon request from the corresponding author.

## Supplementary Materials

The following supporting information can be downloaded at: https://www.mdpi.com/article/10.3390/pharmaceutics14081745/s1, Table S1: Drug to polymer ratio (*w/w*) effect on the physicochemical properties, encapsulation efficiency (EE%) and drug loading of ATRA-PLGA NPs using nanoprecipitation method; nanoparticles were formulated using Resomer RG 752H—PLGA and were loaded with different drug to polymer ratios (*w/w*). All loaded formulations were purified using centrifugation, disrupted in 1:100 (*v/v*) NPs/MeOH. Drug content was quantified by UV-Vis at 348 nm. Hydrodynamic size (Z-average), Polydispersity index (PDI) and zeta potential of NPs were measured by the Malvern Nanosizer ZS90. Data shown as mean ± SD (n = 3). Figure S1: Effect of nebulization on the physicochemical properties (A) size, (B) PDI of ATRA-PLGA NPs suspended in water; Nanoparticles were nebulized into 50 mL falcon tube and then samples were collected. The average hydrodynamic size and PDI NPs were measured by the Malvern Nanosizer ZS90. Statistical analysis was done using student’s *t*-test. Data shown as mean ± SD (n = 3).
