# Supplementary material for: Development of Inhalable ATRA-Loaded PLGA Nanoparticles as Host-Directed Immunotherapy against Tuberculosis
Source: Pharmaceutics. 2022 Aug 21;14(8):1745. doi: 10.3390/pharmaceutics14081745 (PMC9415714; doi:10.3390/pharmaceutics14081745)
Supplement: Supplementary file 1 [file pharmaceutics-14-01745-s001.zip › pharmaceutics-1859930-supplementary.pdf]

Supplementary material

Supplementary Table S1: Drug to polymer ratio (w/w) effect on the physicochemical properties, encapsulation efficiency (EE%)and drug loading of ATRA-PLGA NPs using nanoprecipitation method; nanoparticles were formulated using Resomer RG 752H - PLGA and were loaded with different drug to polymer ratios (w/w). All loaded formulations were purified using centrifugation, disrupted in 1:100 (v/v) NPs/MeOH. Drug content was quantified by UV-Vis at 348 nm. Hydrodynamic size (Z-average), Polydispersity index (PDI) and zeta potential of NPs were measured by the Malvern Nanosizer ZS90. Data shown as mean ± SD (n = 3).

**Drug to polymer (w/w)**	**Z-average (nm)**	**PDI**	**Zeta potential (mV)**	**EE%**	**Drug loading (µg/mg)**
Blank 0:10	173.2 ± 1.7	0.035 ± 0.018	- 1.27 ± 0.016	N/A	N/A
Blank 0:20	203.9 ± 0.4	0.041 ± 0.006	- 2.52 ± 0.331	N/A	N/A
0.1:10	200.0 ± 8.8	0.359 ± 0.060	-1.64 ± 0.134	39.6 ± 22.5	4.7 ± 2.12
0.5:10	264.1 ± 5.1	0.265 ± 0.001	-1.40 ± 0.040	69.8 ± 0.53	40.2 ± 7.56
1:10	321.8 ± 11.4	0.281 ± 0.130	-2.18 ± 1.320	53.7 ± 13.8	53.9 ± 13.93
0.5:20	235.4 ± 0.5	0.168 ± 0.008	-1.71 ± 0.200	57.6 ± 9.75	15.9 ± 2.61
1:20	261.6 ± 9.7	0.187 ± 0.030	-1.80 ± 0.410	65.8 ± 12.4	33.8 ± 4.56
2:20	287.4 ± 13.3	0.205 ± 0.033	-1.98 ± 0.660	64.8 ± 15.4	55.8 ± 0.42

A)

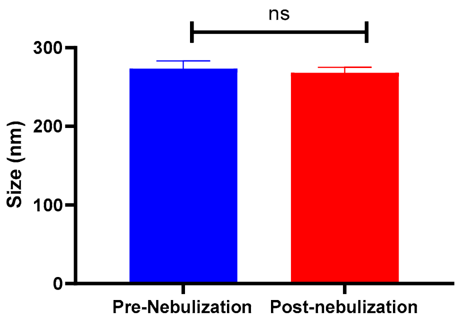

B)

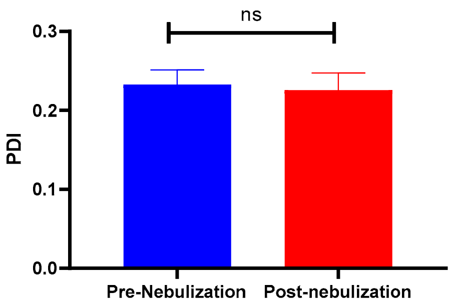

Supplementary Figure S1: Effect of nebulization on the physicochemical properties A) size, B) PDI of ATRA-PLGA NPs suspended in water ; Nanoparticles were nebulized into 50 mL falcon tube and then samples were collected. The average hydrodynamic size and PDI NPs were measured by the Malvern Nanosizer ZS90. Statistical analysis was done using student's t-test test. Data shown as mean ± SD (n = 3).
